# Supplementary figures and images for: Development and implementation of a simple and rapid extraction-free saliva SARS-CoV-2 RT-LAMP workflow for workplace surveillance
Source: PLoS One. 2022 May 26;17(5):e0268692. doi: 10.1371/journal.pone.0268692 (PMC9135294; doi:10.1371/journal.pone.0268692)

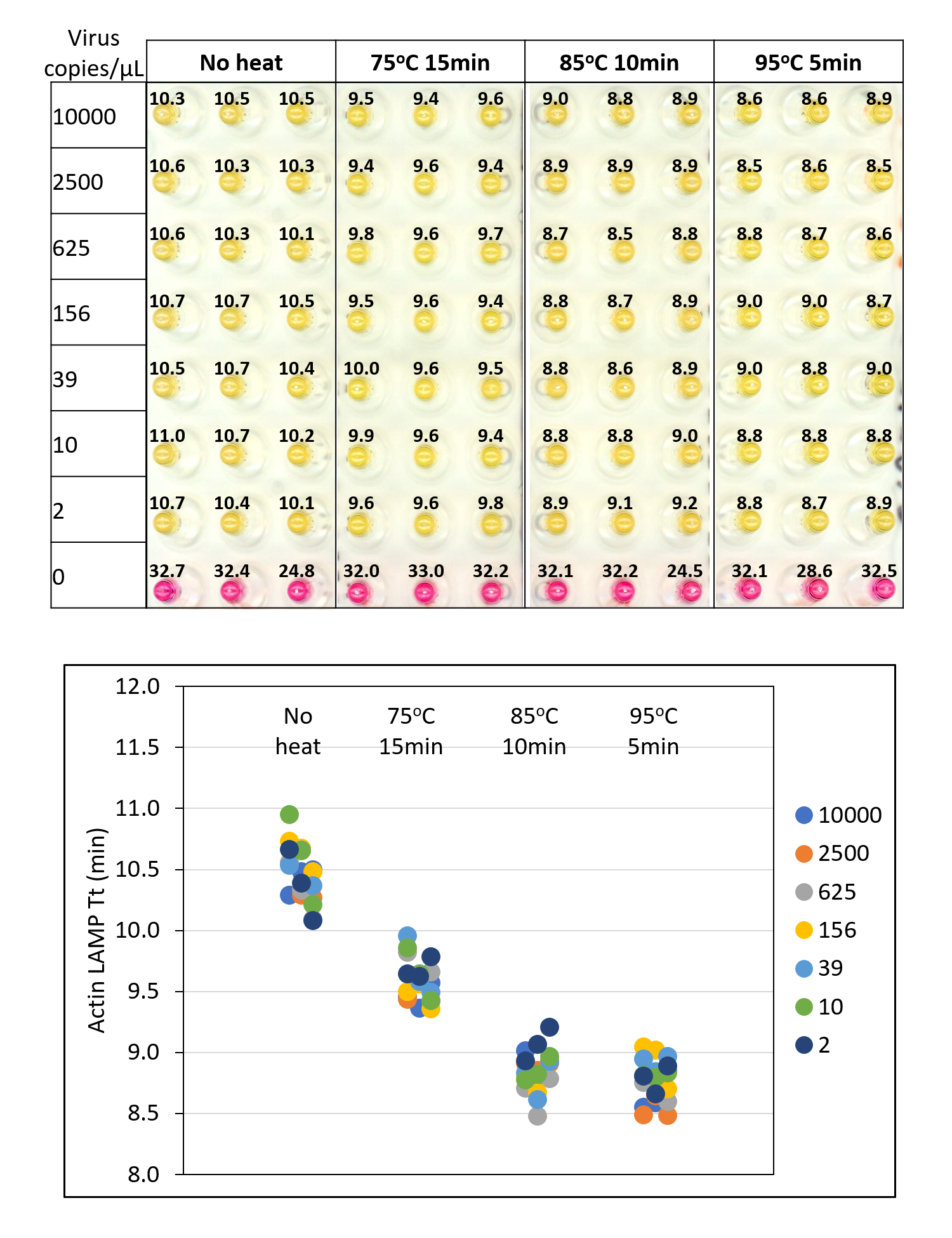

Supplement: S1 Fig — Contrived samples spiked with 2–10,000 viral copies/μL, were processed in triplicate using either 75°C for 15 min, 85°C for 10 min, 95°C for 5 min, or no heat. The scanned image of the post-amplification actin LAMP plate with overlaid Tt values (upper panel) and the plot of actin Tt values (lower panel) are shown. (TIF) [file pone.0268692.s001.tif]

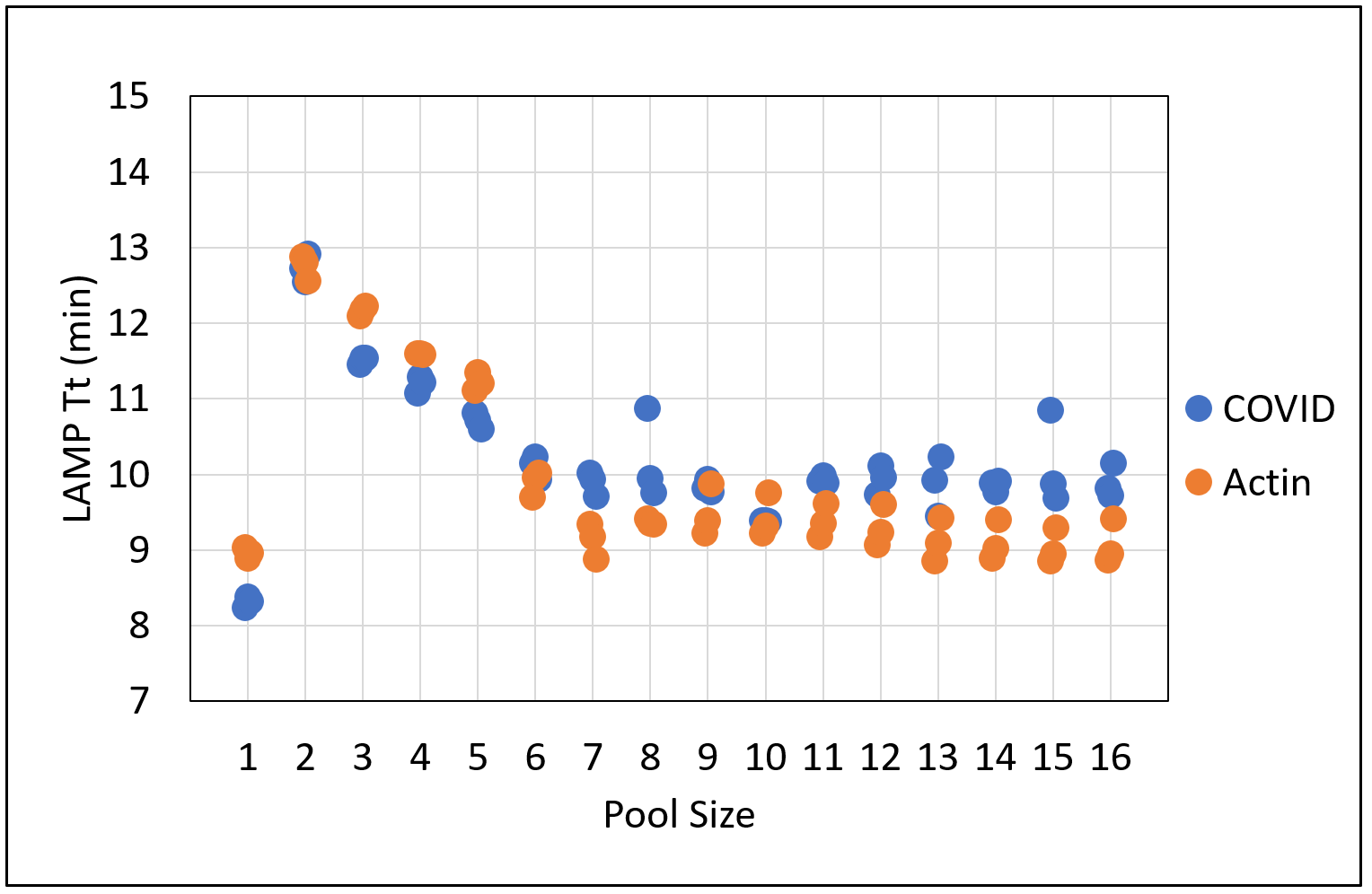

Supplement: S2 Fig — Positive saliva was combined with equal volumes of 1 to 15 randomly selected negative saliva samples from different individuals, corresponding to dilution series of 1:2 through 1:16 of the original viral titer, and tested in COVID and actin LAMP reactions. All samples were tested in triplicate. LAMP Tt values are plotted. (TIF) [file pone.0268692.s002.tif]

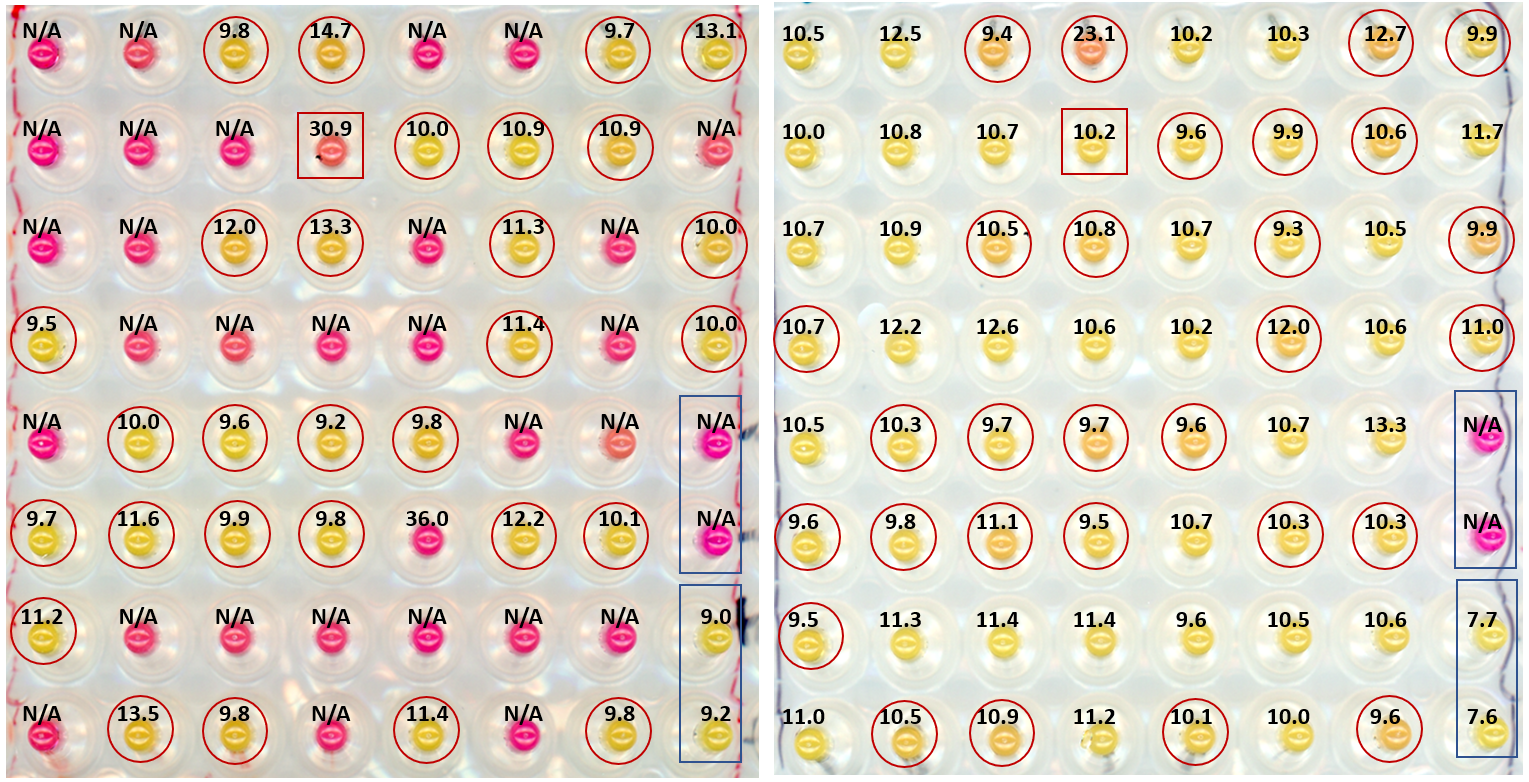

Supplement: S3 Fig — Previously identified SARS-CoV-2 positive (n = 30) and negative (n = 30) samples were tested blindly in COVID and actin LAMP reactions. The scanned images of the post-amplification COVID and actin LAMP plates are shown with overlaid Tt values. Positive samples were revealed after the experiment and marked with red circles. The missed positive sample was marked with a red square. Negative and positive controls are marked with a blue rectangle. Experiments were performed 3 times with 2 different operators. The result from one experiment is shown. (TIF) [file pone.0268692.s003.tif]

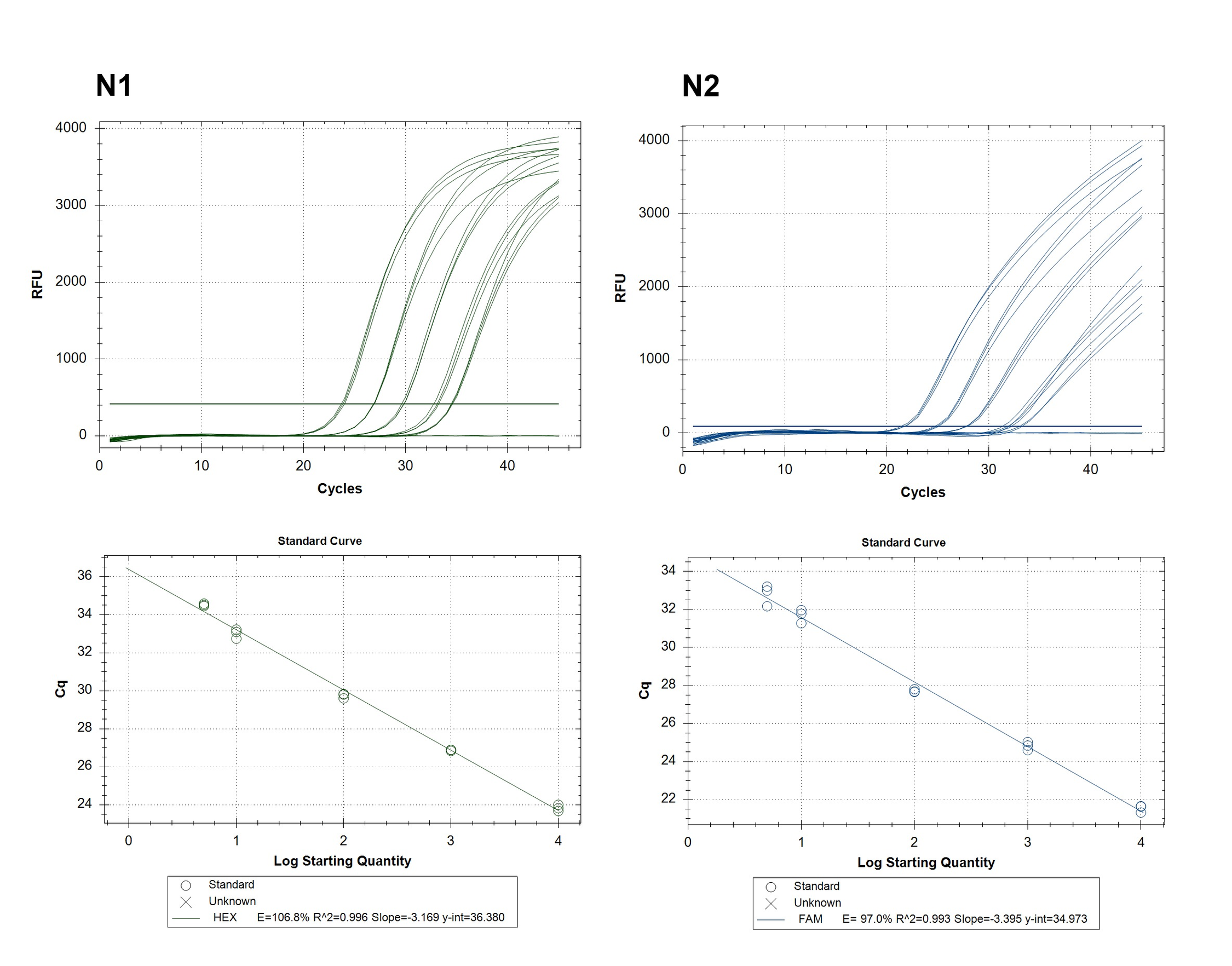

Supplement: S4 Fig — Different copy numbers of Twist RNA (5, 10, 100, 1000, and 10000) were used to generate a standard curve using the Luna® SARS-CoV-2 RT-qPCR Multiplex Assay Kit. Amplification and standard curves from both N1 and N2 targets are shown. (TIF) [file pone.0268692.s004.tif]

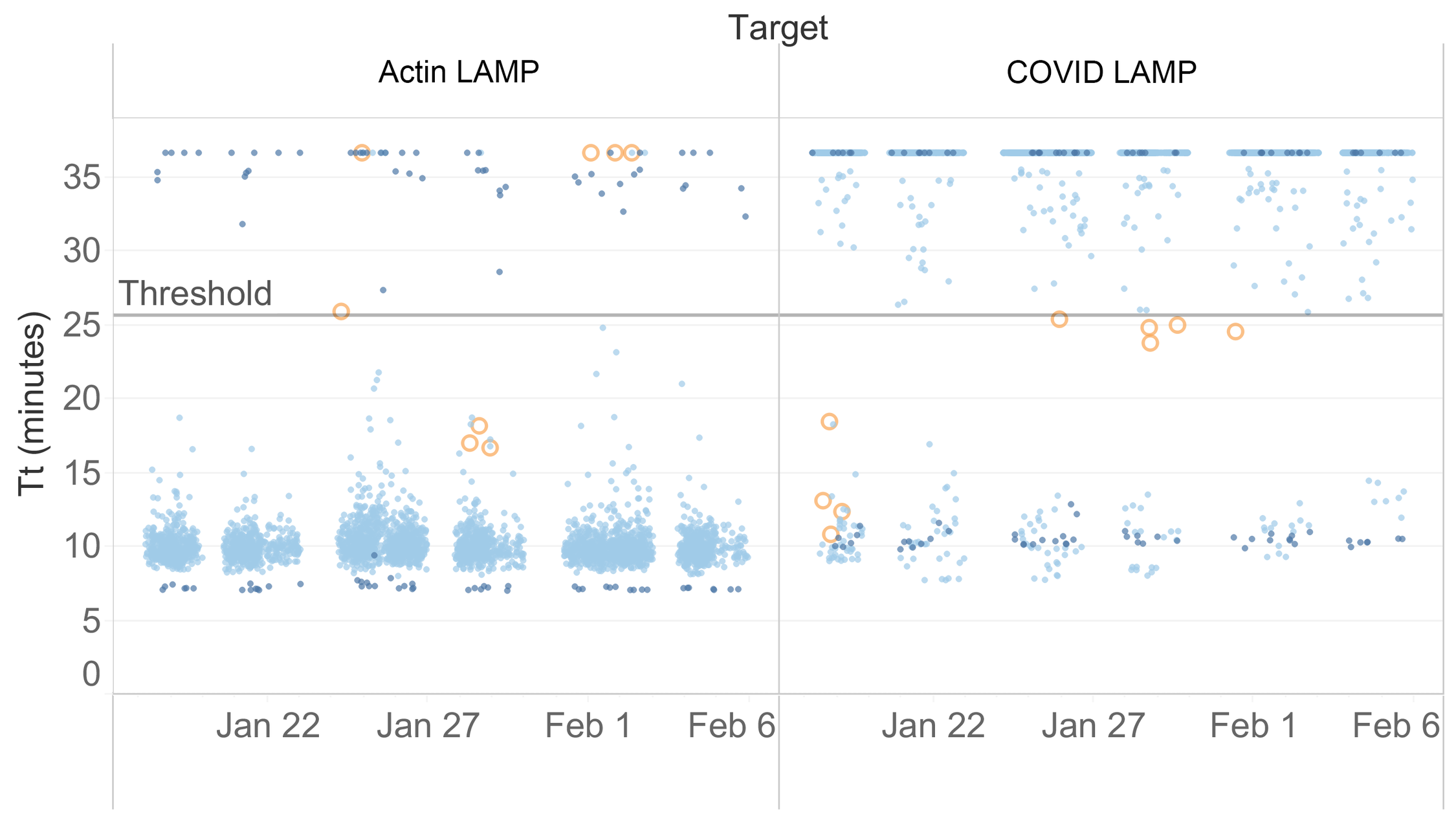

Supplement: S6 Fig — 3,654 samples were evaluated. The testing date (X axis) and the Tt values from COVID and actin LAMP assays are plotted (Y axis). A Tt value of 36 minutes was assigned to reactions with no fluorescent signal detected (N/A). The post-amplification color images were scored. Light blue dots represent samples that generated a concordant result. Discordant samples are shown with orange circles. All control reactions represented by dark blue dots were concordant. (TIF) [file pone.0268692.s006.tif]

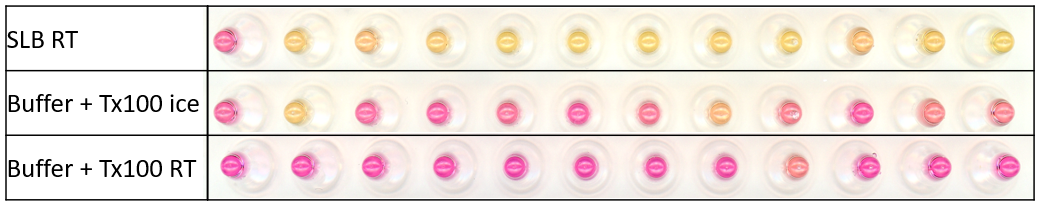

Supplement: S7 Fig — Contrived saliva samples spiked with 20 viral copies/μL were mixed with SLB buffer or Cepko buffer with 0.2% Triton X-100. Samples were incubated on ice or at room temperature for 20 minutes before being heated at 95°C for 5min. Covid LAMP reactions were performed with 2μL of lysate. Post amplification image of 12 reactions per condition was shown. Positive reactions showed yellow color and negative reactions showed pink/orange color. (TIF) [file pone.0268692.s007.tif]

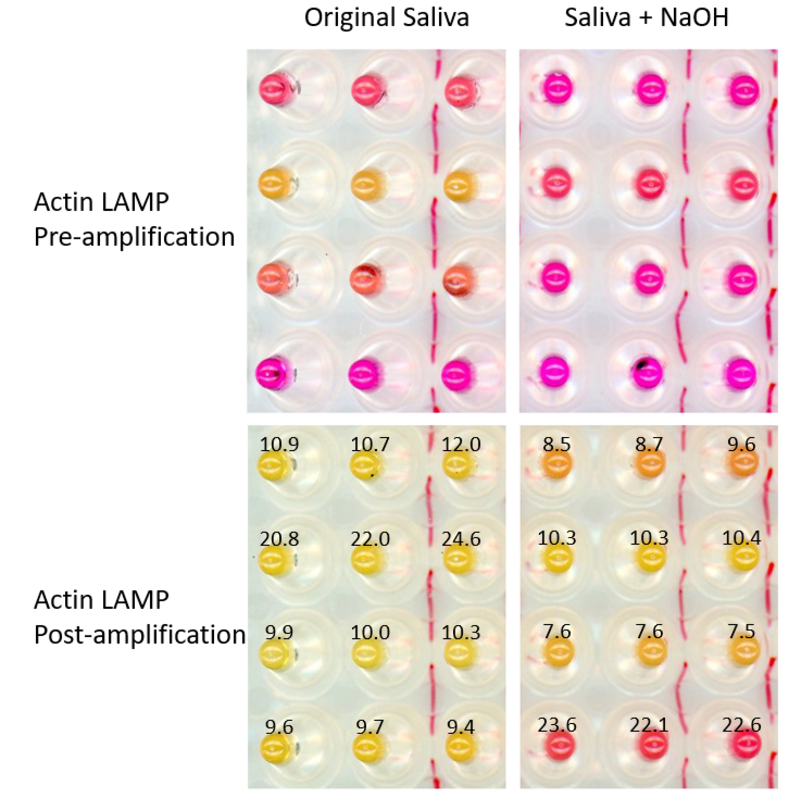

Supplement: S8 Fig — Four different saliva samples were tested (triplicate) in actin LAMP reaction with or without 30mM of NaOH. The pre-amplification image and post-amplification image overlaid with Tt value are shown. (TIF) [file pone.0268692.s008.tif]

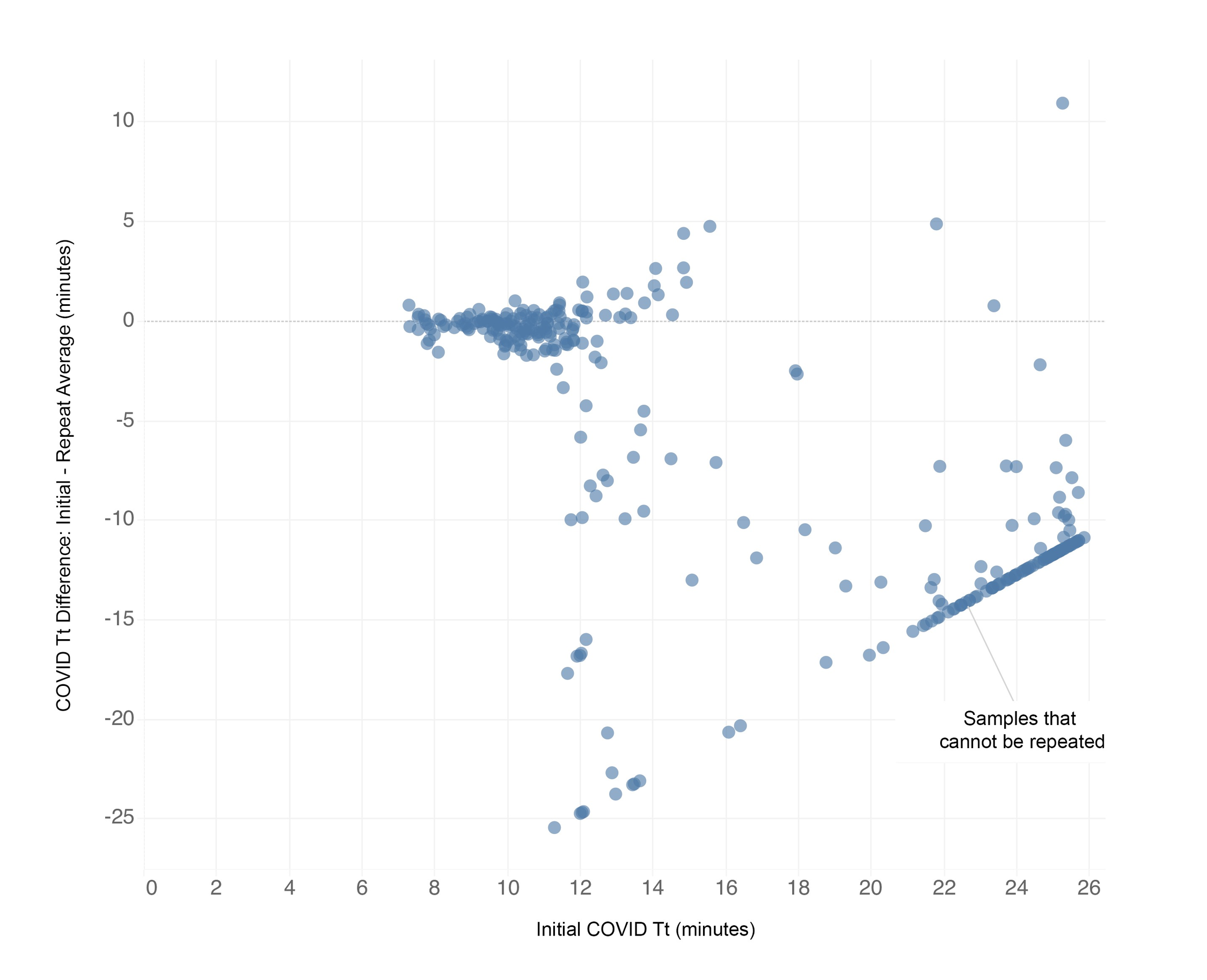

Supplement: S9 Fig — All samples which generated a COVID Tt value below 26 minutes were repeated in triplicate. The initial COVID Tt value (X axis) and Tt difference between the initial and the average of triplicate repeat tests (Y axis) were plotted. Each dot represents a single sample. Tt value of 36 minutes was assigned to reactions with no fluorescent signal detected (N/A) for calculation purposes. (TIF) [file pone.0268692.s009.tif]
