# Supplementary material for: Development and implementation of a simple and rapid extraction-free saliva SARS-CoV-2 RT-LAMP workflow for workplace surveillance
Source: PLoS One. 2022 May 26;17(5):e0268692. doi: 10.1371/journal.pone.0268692 (PMC9135294; doi:10.1371/journal.pone.0268692)
Supplement: S5 Fig — (A) User Interface: (1) Patient Log In, (2) Sample Submission and (3) Patient Result. (B) Operator Interface: (1) New Run, (2) Results Uploaded, (3) Sample Status, (4) Results Review and (5) Batch History. (PDF) [file pone.0268692.s005.pdf]

Figure S5. Electronic Health Records Portal: cov.neb.com

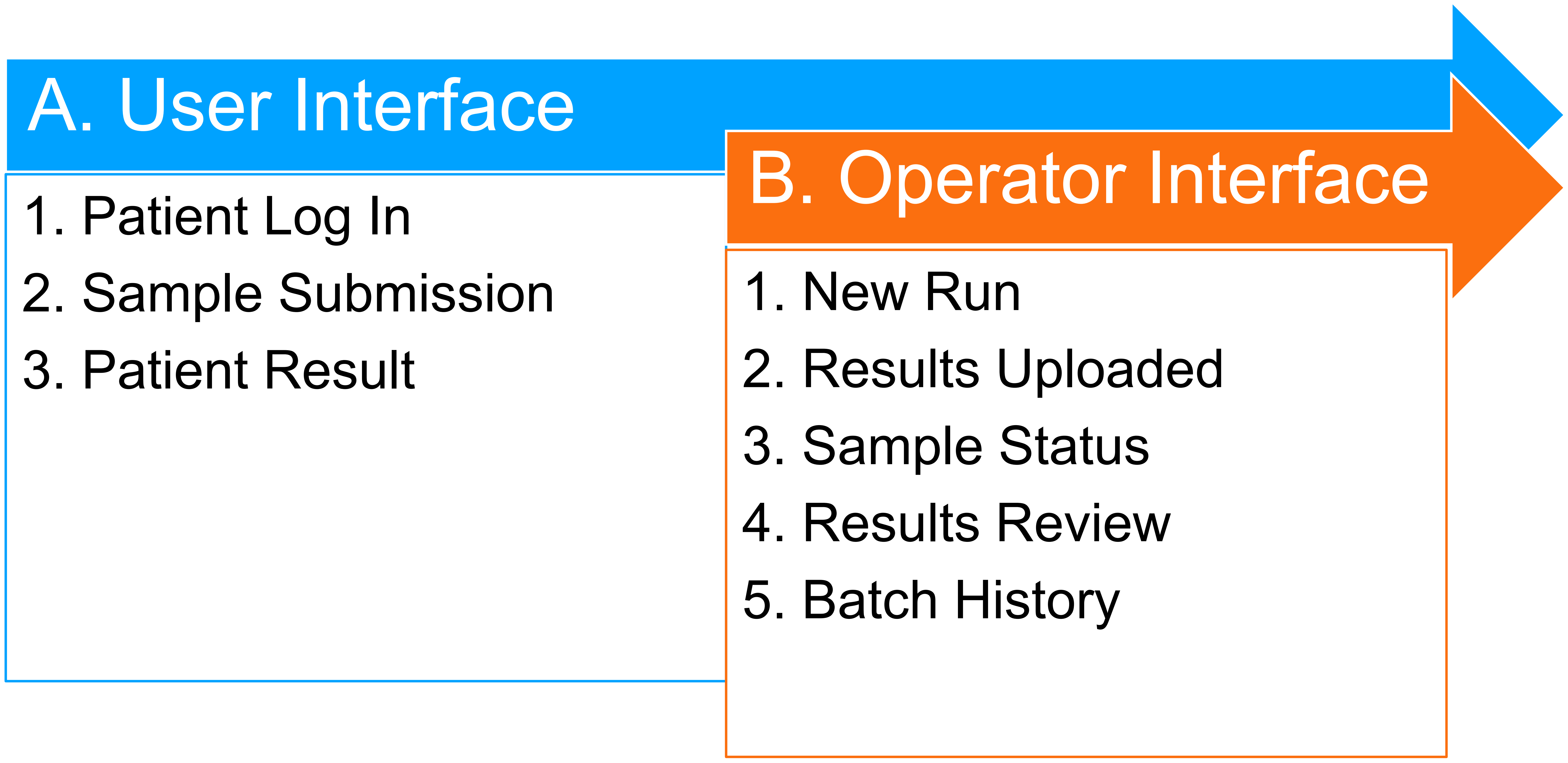

A. User Interface

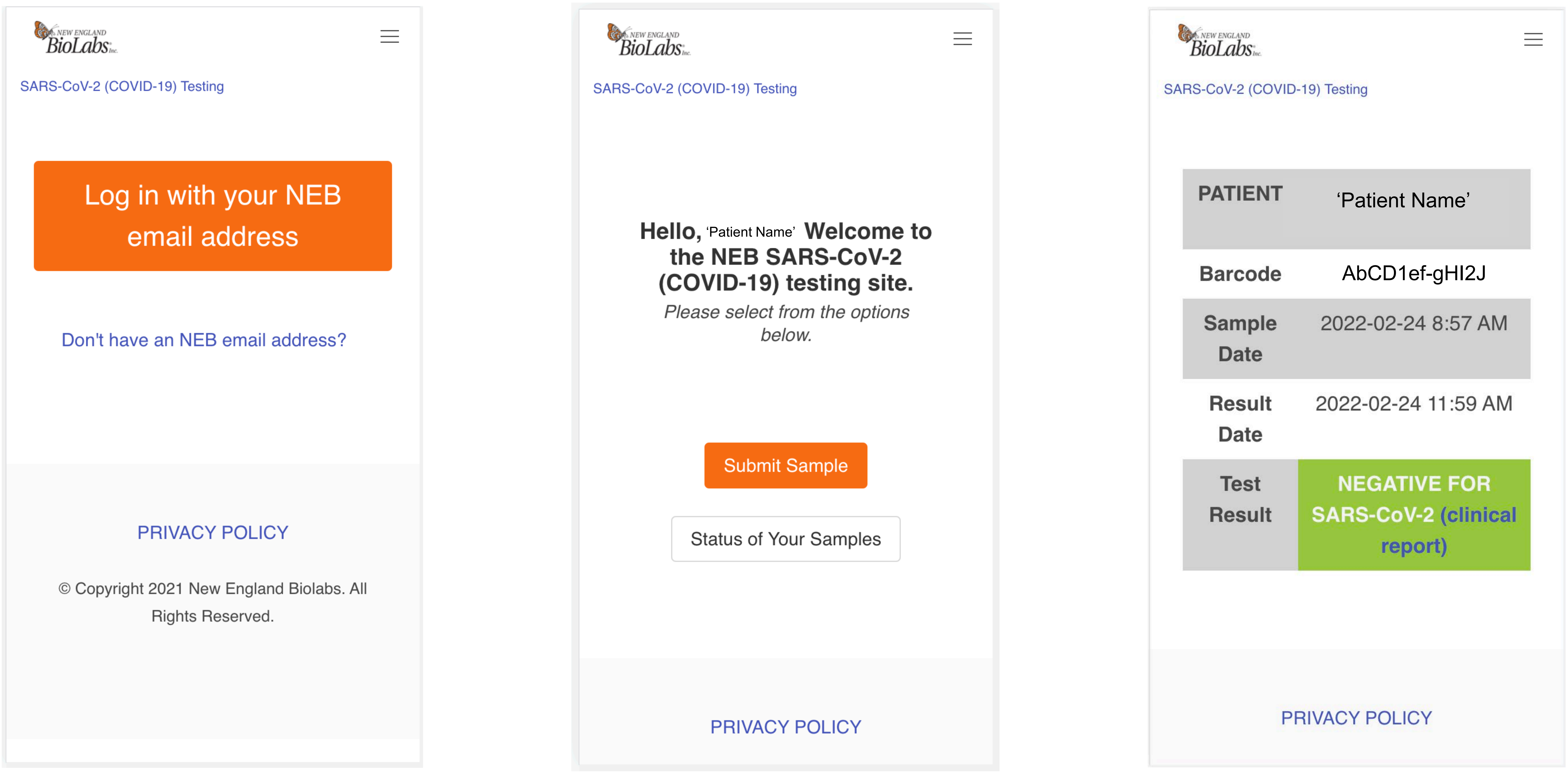

B. Operator Interface

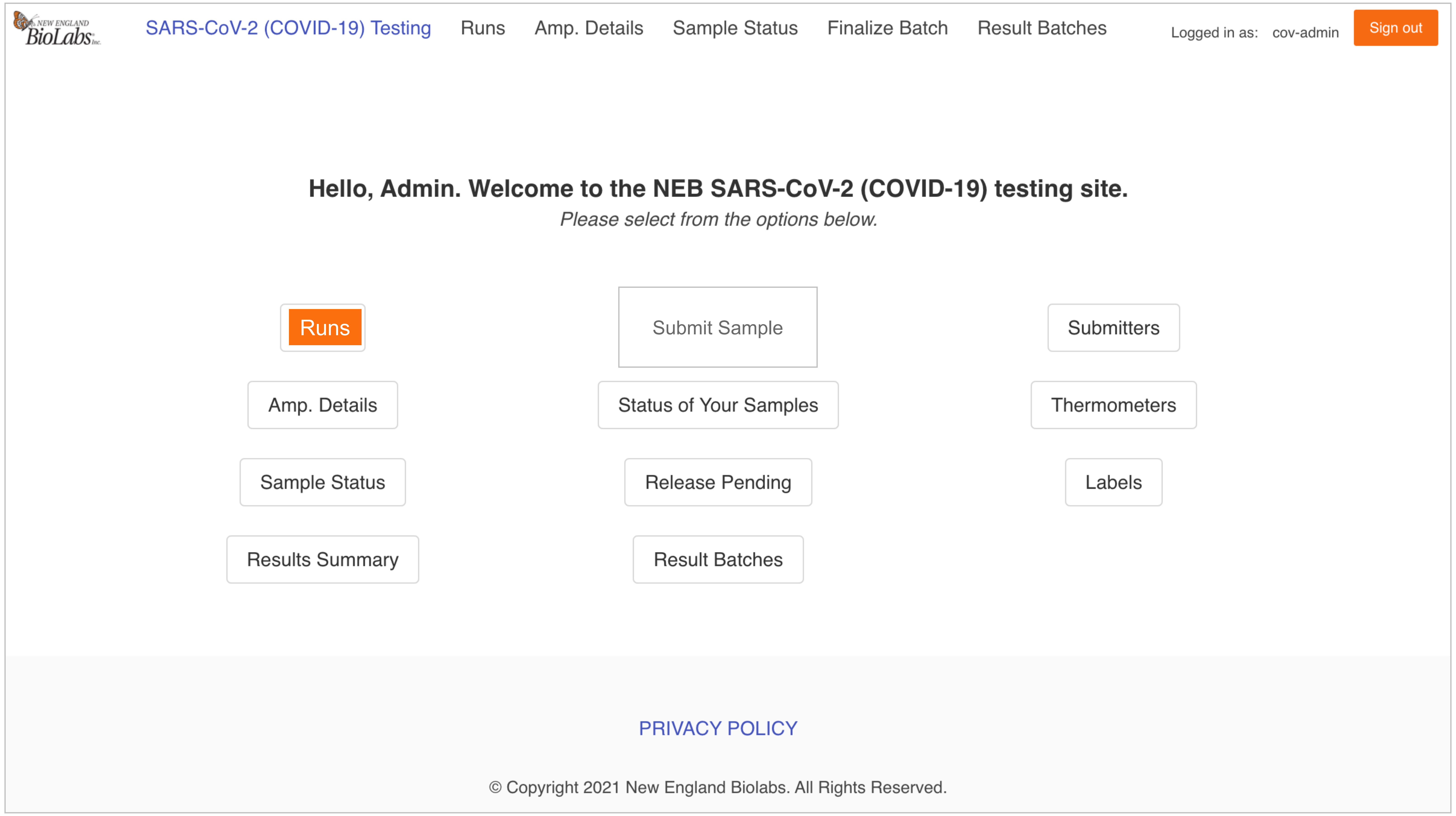

B1. New Run

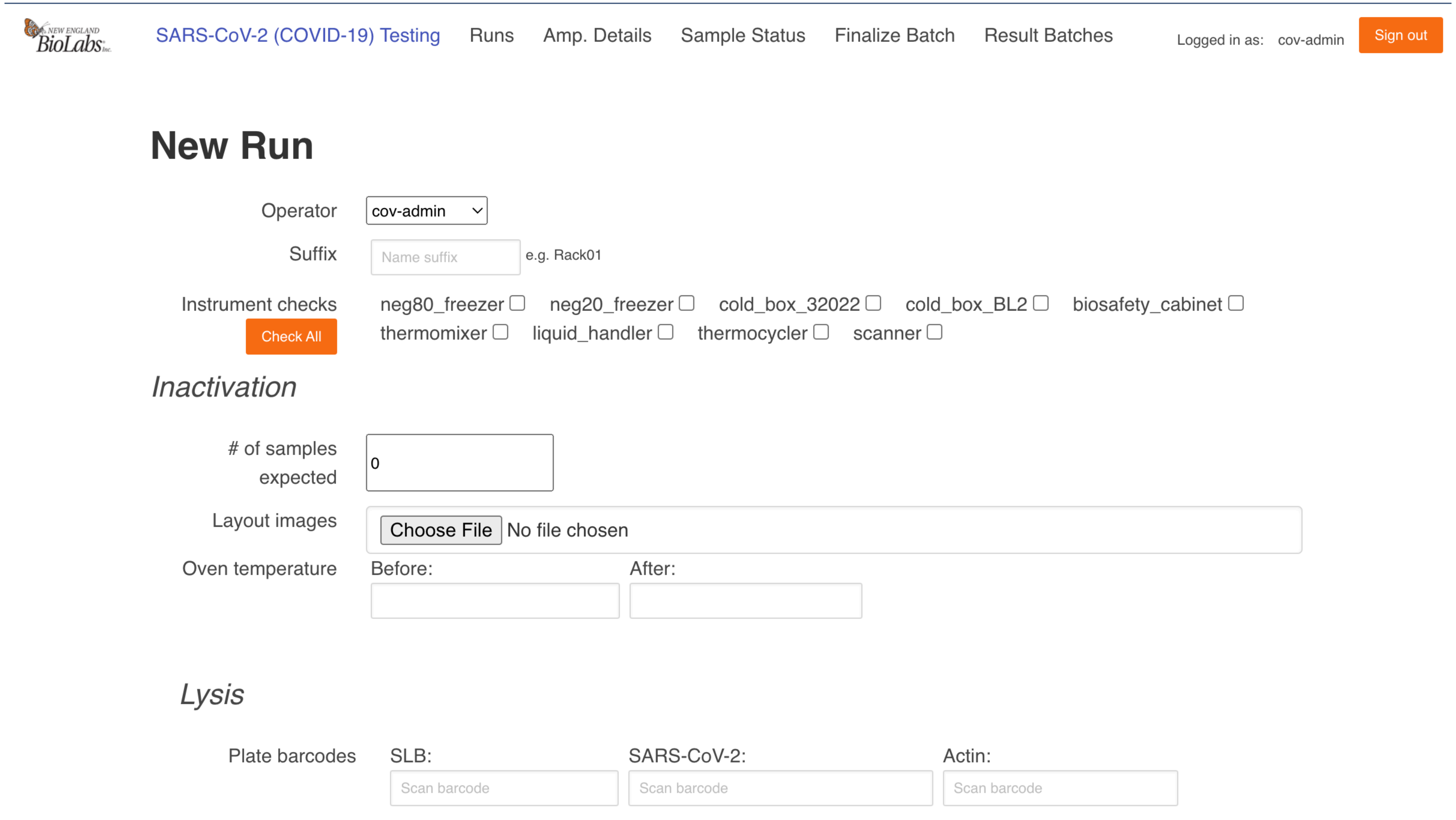
NEW ENGLAND BioLabs Inc.
SARS-CoV-2 (COVID-19) Testing
Runs
Amp. Details
Sample Status
Finalize Batch
Result Batches
Logged in as: cov-admin
Sign out
New Run
Operator
cov-admin
Suffix
Name suffix
e.g. Rack01
Instrument checks
Check All
neg80_freezer
neg20_freezer
cold_box_32022
cold_box_BL2
biosafety_cabinet
thermomixer
liquid_handler
thermocycler
scanner
Inactivation
# of samples expected
0
Layout images
Choose File
No file chosen
Oven temperature
Before:
After:
Lysis
Plate barcodes
SLB:
SARS-CoV-2:
Actin:
Scan barcode
Scan barcode
Scan barcode

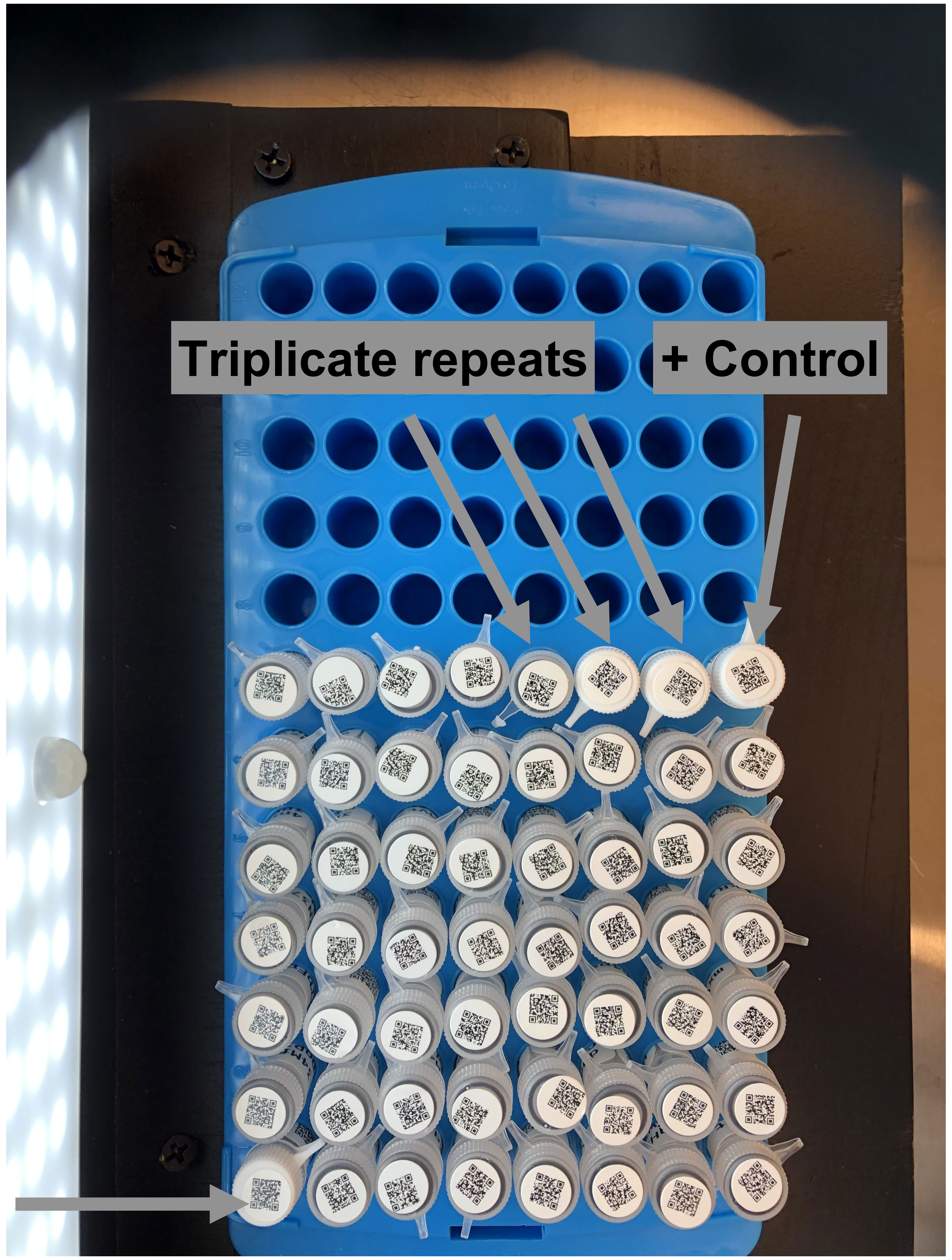
Triplicate repeats
+ Control
- Control

B2. Results Upload

Amplification

Bio-Rad Plate Map Bio-Rad plate map

Plate Barcode 6759475320

Target SARS-CoV-2 N2+E1

Operator rmoncion

Bio-Rad .pcrd file Choose File No file chosen Current .pcrd File

Cq Results .xlsx file Choose File No file chosen Current Cq File

Amp Results .xlsx file Choose File No file chosen Current Amp Results File

Plate Images

Pre-Amp. Image Choose File No file chosen Current Pre-Amp Image

Post-Amp. Image Choose File No file chosen Current Post-Amp Image

Update Bio rad experiment

Destroy | Run

B3. Sample Status

NEW ENGLAND BioLabs Inc. | SARS-CoV-2 (COVID-19) Testing | Runs | Amp. Details | Sample Status | Finalize Batch | Result Batches | Logged in as: cov-admin | Sign out

Samples

All Incomplete | New | In Progress | Final Release Pending | Invalid | Not Received

Check All 17 samples match status: New

Show 100 entries Copy Search:

Sample Date	Sample	Experiments	SARS Cqs	Human Cqs	Result	Release Result	Notes
2022-03-01 9:20 AM	AbCD1ef-gHI2J (New)				Result Pending	no data	Notes...
2022-03-01 11:01 AM	AbCD2ef-gHI2J (New)				Result Pending	no data	Notes...
2022-03-01 12:05 PM	AbCD3ef-gHI2J (New)				Result Pending	no data	Notes...

B4. Result Review

SARS-CoV-2 (COVID-19) Testing | Runs | Amp. Details | Sample Status | Finalize Batch | Result Batches | Logged in as: cov-admin | Sign out

Release Pending Results

Temperature log: Choose File Temperature logs (107).xlsx

Uncheck All

Show 1,000 entries Copy

Search:

Sample Registration Date	Sample	SARS-CoV-2 Cq Values	Human Cq Values	Result	Confirm	Notes
2022-03-02 1:11 PM	AbCD1ef-gHI2J	N/A	27.3	not_detected	☑	
2022-03-02 3:56 PM	AbCD2ef-gHI2J	N/A	25.3	not_detected	☑	
2022-03-02 3:58 PM	AbCD3ef-gHI2J	N/A	25.6	not_detected	☑	
2022-03-02 4:03 PM	AbCD4ef-gHI2J	N/A	28.0	not_detected	☑	

B5. Batch History

SARS-CoV-2 (COVID-19) Testing Runs Amp. Details Sample Status Finalize Batch Result Batches Logged in as: cov-admin Sign out

SARS-CoV-2 Testing Results

Show 10 entries Copy Search:

Batch Approval Date	Result Summary
2022-03-03	SARS_CoV-2 detected in 1/442 samples (0 inconclusive, 3 not identified)
2022-03-01	SARS_CoV-2 detected in 1/418 samples (0 inconclusive, 1 not identified)
2022-02-28	SARS_CoV-2 detected in 0/434 samples (0 inconclusive, 3 not identified)
2022-02-24	SARS_CoV-2 detected in 1/415 samples (0 inconclusive, 4 not identified)
2022-02-22	SARS_CoV-2 detected in 0/368 samples (0 inconclusive, 1 not identified)
2022-02-18	SARS_CoV-2 detected in 0/68 samples (0 inconclusive, 0 not identified)
2022-02-17	SARS_CoV-2 detected in 1/423 samples (0 inconclusive, 5 not identified)
2022-02-15	SARS_CoV-2 detected in 2/407 samples (0 inconclusive, 2 not identified)
2022-02-14	SARS_CoV-2 detected in 1/411 samples (0 inconclusive, 4 not identified)
