# Supplementary material for: Development and implementation of a simple and rapid extraction-free saliva SARS-CoV-2 RT-LAMP workflow for workplace surveillance
Source: PLoS One. 2022 May 26;17(5):e0268692. doi: 10.1371/journal.pone.0268692 (PMC9135294; doi:10.1371/journal.pone.0268692)
Supplement: S1 Table — 16 saliva samples containing a range of viral load were tested in RT-qPCR (RNaseP) and RT-LAMP (Actin). For RT-LAMP, both saliva lysate and purified RNA were used as input, whereas RT-qPCR was performed using only purified RNA. Cq values for RT-qPCR and Tt for RT-LAMP are shown. No amplification is denoted N/A. (DOCX) [file pone.0268692.s010.docx]

**S1 Table. Internal actin control detection of the 16 samples**

|  | **Purified Saliva RNA** | | | | **Saliva Lysate** | |
| --- | --- | --- | --- | --- | --- | --- |
| **Samples** | **RT-qPCR RNase P**  **(Cq)** | | **Actin LAMP**  **(Tt)** | | **Actin LAMP**  **(Tt)** | |
| **1** | N/A | N/A | 8.9 | 9.3 | 9.1 | 9.1 |
| **2** | 43.1 | 43.2 | 10.4 | 10.3 | 11.1 | 11.2 |
| **3** | 30.4 | 30.2 | 7.2 | 7.2 | 8.8 | 8.8 |
| **4** | 33.9 | 39.5 | 9.2 | 9.3 | 10.6 | 10.6 |
| **5** | 41.1 | 39.0 | 8.3 | 8.3 | 11.1 | 11.4 |
| **6** | 35.1 | 34.8 | 8.5 | 8.7 | 12.1 | 12.0 |
| **7** | 35.4 | 35.9 | 9.4 | 9.4 | 10.2 | 10.3 |
| **8** | 33.7 | 33.2 | 8.3 | 8.3 | 11.8 | 11.9 |
| **9** | 40.1 | N/A | 10.8 | 11.5 | 10.7 | 10.7 |
| **10** | N/A | 38.0 | 11.0 | 11.8 | 10.6 | 10.4 |
| **11** | 39.9 | 37.3 | 9.1 | 9.0 | 9.4 | 9.9 |
| **12** | 33.2 | 33.7 | 9.7 | 9.6 | 9.6 | 9.7 |
| **13** | 33.7 | 33.7 | 8.4 | 8.5 | 10.3 | 10.2 |
| **14** | 37.7 | 38.9 | 10.8 | 11.1 | 10.6 | 10.0 |
| **15** | 32.8 | 32.9 | 8.5 | 8.5 | 10.0 | 10.0 |
| **16** | 38.8 | 38.8 | 9.8 | 10.2 | 10.5 | 10.5 |

16 saliva samples containing a range of viral load were tested in RT-qPCR (RNaseP) and RT-LAMP (Actin). For RT-LAMP, both saliva lysate and purified RNA were used as input, whereas RT-qPCR was performed using only purified RNA. Cq values for RT-qPCR and Tt for RT-LAMP are shown. No amplification is denoted N/A.
