# Supplementary material for: Development and implementation of a simple and rapid extraction-free saliva SARS-CoV-2 RT-LAMP workflow for workplace surveillance
Source: PLoS One. 2022 May 26;17(5):e0268692. doi: 10.1371/journal.pone.0268692 (PMC9135294; doi:10.1371/journal.pone.0268692)
Supplement: Peer review history

# Peer review: Decision Letter 0

**DOI:** 10.1371/journal.pone.0268692.r001

**Authors:** Ruslan Kalendar

6 Apr 2022

PONE-D-22-07620Development and Implementation of a Simple and Rapid Extraction-Free Saliva SARS-CoV-2 RT-LAMP Workflow for Workplace SurveillancePLOS ONE

Dear Dr. Carlow,

Thank you for submitting your manuscript to PLOS ONE. After careful consideration, we feel that it has merit but does not fully meet PLOS ONE’s publication criteria as it currently stands. Therefore, we invite you to submit a revised version of the manuscript that addresses the points raised during the review process.

Please submit your revised manuscript by May 21 2022 11:59PM. If you will need more time than this to complete your revisions, please reply to this message or contact the journal office at plosone@plos.org. When you're ready to submit your revision, log on to https://www.editorialmanager.com/pone/ and select the 'Submissions Needing Revision' folder to locate your manuscript file.

Please include the following items when submitting your revised manuscript:

- A rebuttal letter that responds to each point raised by the academic editor and reviewer(s). You should upload this letter as a separate file labeled 'Response to Reviewers'.
- A marked-up copy of your manuscript that highlights changes made to the original version. You should upload this as a separate file labeled 'Revised Manuscript with Track Changes'.
- An unmarked version of your revised paper without tracked changes. You should upload this as a separate file labeled 'Manuscript'.

If you would like to make changes to your financial disclosure, please include your updated statement in your cover letter. Guidelines for resubmitting your figure files are available below the reviewer comments at the end of this letter.

If applicable, we recommend that you deposit your laboratory protocols in protocols.io to enhance the reproducibility of your results. Protocols.io assigns your protocol its own identifier (DOI) so that it can be cited independently in the future. For instructions see: https://journals.plos.org/plosone/s/submission-guidelines#loc-laboratory-protocols. Additionally, PLOS ONE offers an option for publishing peer-reviewed Lab Protocol articles, which describe protocols hosted on protocols.io. Read more information on sharing protocols at https://plos.org/protocols?utm_medium=editorial-email&utm_source=authorletters&utm_campaign=protocols.

We look forward to receiving your revised manuscript.

Kind regards,

Ruslan Kalendar

Academic Editor

PLOS ONE

Journal Requirements:

When submitting your revision, we need you to address these additional requirements.

1. Please ensure that your manuscript meets PLOS ONE's style requirements, including those for file naming. The PLOS ONE style templates can be found at 

https://journals.plos.org/plosone/s/file?id=wjVg/PLOSOne_formatting_sample_main_body.pdf and 

https://journals.plos.org/plosone/s/file?id=ba62/PLOSOne_formatting_sample_title_authors_affiliations.pdf

2. Thank you for stating the following in the Competing Interests section: 

"New England Biolabs (www.neb.com) has funded this study. All authors (ZL,JLB, BC, CVC, WEJ, KK, BWL, JB, RAM, CBP, GR, RJR, NAT, YZ and CKSC) are employees of New England Biolabs, manufacturer of LAMP and PCR reagents described in the manuscript."  

We note that you received funding from a commercial source: New England Biolabs

Please provide an amended Competing Interests Statement that explicitly states this commercial funder, along with any other relevant declarations relating to employment, consultancy, patents, products in development, marketed products, etc. 

Within this Competing Interests Statement, please confirm that this does not alter your adherence to all PLOS ONE policies on sharing data and materials by including the following statement: "This does not alter our adherence to PLOS ONE policies on sharing data and materials.” (as detailed online in our guide for authors http://journals.plos.org/plosone/s/competing-interests).  If there are restrictions on sharing of data and/or materials, please state these. Please note that we cannot proceed with consideration of your article until this information has been declared. 

Please include your amended Competing Interests Statement within your cover letter. We will change the online submission form on your behalf.

3.Thank you for stating the following in the Acknowledgments Section of your manuscript: 

"This work was supported by New England Biolabs."

We note that you have provided funding information that is not currently declared in your Funding Statement. However, funding information should not appear in the Acknowledgments section or other areas of your manuscript. We will only publish funding information present in the Funding Statement section of the online submission form. 

Please remove any funding-related text from the manuscript and let us know how you would like to update your Funding Statement. Currently, your Funding Statement reads as follows: 

"New England Biolabs (www.neb.com) has funded this study. ZL, JLB, BC, CVC, WEJ, KK, BWL, JM, RAM, CBP, GR, RJR, NAT, YZ and CKSC are employees of New England Biolabs. The funders had no role in study design, data collection and analysis, decision to publish, or preparation of the manuscript."

Please include your amended statements within your cover letter; we will change the online submission form on your behalf.

4. We note that you have included the phrase “data not shown” in your manuscript. Unfortunately, this does not meet our data sharing requirements. PLOS does not permit references to inaccessible data. We require that authors provide all relevant data within the paper, Supporting Information files, or in an acceptable, public repository. Please add a citation to support this phrase or upload the data that corresponds with these findings to a stable repository (such as Figshare or Dryad) and provide and URLs, DOIs, or accession numbers that may be used to access these data. Or, if the data are not a core part of the research being presented in your study, we ask that you remove the phrase that refers to these data.

5. Please review your reference list to ensure that it is complete and correct. If you have cited papers that have been retracted, please include the rationale for doing so in the manuscript text, or remove these references and replace them with relevant current references. Any changes to the reference list should be mentioned in the rebuttal letter that accompanies your revised manuscript. If you need to cite a retracted article, indicate the article’s retracted status in the References list and also include a citation and full reference for the retraction notice.

Reviewers' comments:

Reviewer's Responses to Questions

**Comments to the Author**

1. Is the manuscript technically sound, and do the data support the conclusions?

The manuscript must describe a technically sound piece of scientific research with data that supports the conclusions. Experiments must have been conducted rigorously, with appropriate controls, replication, and sample sizes. The conclusions must be drawn appropriately based on the data presented. 

Reviewer #1: Yes

Reviewer #2: Yes

**********

2. Has the statistical analysis been performed appropriately and rigorously? 

Reviewer #1: Yes

Reviewer #2: Yes

**********

3. Have the authors made all data underlying the findings in their manuscript fully available?

The PLOS Data policy requires authors to make all data underlying the findings described in their manuscript fully available without restriction, with rare exception (please refer to the Data Availability Statement in the manuscript PDF file). The data should be provided as part of the manuscript or its supporting information, or deposited to a public repository. For example, in addition to summary statistics, the data points behind means, medians and variance measures should be available. If there are restrictions on publicly sharing data—e.g. participant privacy or use of data from a third party—those must be specified.

Reviewer #1: Yes

Reviewer #2: Yes

**********

4. Is the manuscript presented in an intelligible fashion and written in standard English?

PLOS ONE does not copyedit accepted manuscripts, so the language in submitted articles must be clear, correct, and unambiguous. Any typographical or grammatical errors should be corrected at revision, so please note any specific errors here.

Reviewer #1: Yes

Reviewer #2: Yes

**********

5. Review Comments to the Author

Reviewer #1:

In this manuscript, the authors have developed and validated a saliva-based nucleic acid amplification test (RT-LAMP) that offers speed and convenience compared to the typical NAAT that is employed for SARS-CoV2 detection, RT-qPCR. The authors optimized a buffer for virus inactivation and RNA stabilization from saliva, and implemented fluorescence-based and colorimetric detection systems for determining the results of the reaction. Authors determined the major validation parameters for a diagnostic test, including analytical sensitivity (not specificity), and diagnostic sensitivity and specificity compared to the RT-qPCR test. The authors employed the test for the purposes of surveillance at a workplace setting, and screened a large number of samples (>30k) over the course of nearly a year. Authors conclude that the RT-LAMP can offer a rapid, convenient, and relatively inexpensive means to monitor staff for infection in order to mitigate workplace spread of covid. Overall, this is a comprehensive and well-written description of a fast and convenient means to detect SARS-CoV2 in clinical samples that can be challenging (saliva), but offer many advantages compared to deep nasal swabs. There is really not much to criticize in this work. I offer below some minor comments and suggestions for improvement.

Materials and Methods

1. Line 187: The authors should mention here that orange-colored reactions (which in my experience tend to occur when target NA levels are low, and the reaction is just beginning to turn positive when the assay is stopped) were considered negative. This is mentioned much later on in the Results section (lines 402, 404, and Table 2), but should be mentioned here for clarity as well.

Results

2. Line 261 and 278 (Figure 2 legend): I suggest that the authors change the title of this section – “diagnostic sensitivity” typically refers to the performance of a newly developed assay compared to a previously established “gold standard” assay, so this is what I was expecting this section to describe. Instead, that comes later. Perhaps something like, “Impact of saliva input on RT-LAMP performance” or something similar might be suitable.

3. Lines 322-323: This section describes the determination of the analytical sensitivity (LOD) of the RT-LAMP reaction. The authors should specify how the LOD was defined – this is typically specified as the C95, or the concentration of analyte that results in a positive reaction in 95% of assays (see Burd EM Clin. Microbiol. Rev. 23, 550-576, doi:10.1128/cmr.00074-09 (2010)). Moreover, the authors seem to have determined LOD empirically, but there are more accurate means of calculating the C95 for which the authors already have the data – eg see Klymus, K. E. et al. Environ. DNA 2, 271-282, doi:https://doi.org/10.1002/edn3.29 (2020). Please consider expressing the analytical sensitivity data using one of the mathematical models described therein, or alternatively using probit analysis to provide a more precise estimate of the LOD of the assay.

4. Lines 339-345: It was nice to see the Dsp and Dsn of the RT-LAMP assay compared to the RT-qPCR assay, although the number of samples analyzes was rather small. The results are overall rather similar to the values obtained using LAMPore on much larger sample sizes (Ptasinska, A. et al. Clinical microbiology and infection 27, 1348.e1341-1348.e1347, doi:10.1016/j.cmi.2021.04.008 (2021). This reference should be cited in the Discussion. The results obtained here are comparable and vastly less expensive compared to LAMPore, which is only reasonable to perform on large numbers of samples and is therefore not suitable for point-of-care applications.

5. Lines 396-406 and Table 2: These results could be analyzed using the kappa statistic, which is ideally suited to assessing assay performance with a subjective aspect like color development. Please consider adding a kappa calculation to demonstrate the operator agreement.

Reviewer #2: 

Li et al. developed an extraction-free, direct RT-LAMP protocol and validated for screening of employees. The purpose and flow of the study is well defined and methods were around technical standard. Modifications that clarify some technical details/objective assessments that are important as a scientific paper are needed before consideration for publication.

The study was funded by a company and all authors belonged to that company. The study focused on the company’s product. In this situation, is it good for the authors to simply declare that the funder had no role in conducting the study?

Line 52. What was the limit of detection of the developed workflow? 50 copies/uL of samples is far less sensitive than the commercially available RT-qPCR-based assays (usually they show LODs around 1 copies/ul).

Line 56-58. This conclusive statement is not based on the presented results. How rapidly were these infected cases diagnosed and isolated? At least describe turn-around time and time to isolation. How was the viral loads of these cases and how many cases were asymptomatic? I recommend the authors to make a conclusive statement for the developed assay, not what is speculated and is limited to workplace surveillance.

Line 119. Please elaborate RNA extraction and RT-qPCR. How many samples were provided?

Line 139. What was the volume of sample used for RNA purification?

Line 163. Which realtime PCR system was used for fluorescence detection?

Line 197-203 is duplicate of line 130-142.

Line 279. Which centrifugation conditions were used?

Line 295. Is 50 copies/ul concentration used for reaction=100 copies/reaction? Please clarify for understanding. If so, this is equevalent to 25 copies/ul=25000 copies/mL of inactivated saliva. Even with this high concentration, 1.4% of samples were tested negative, indicating low sensitivity of the assay and this is supported by LOD analysis.

Line 297. The RT-LAMP reaction was performed according to manufacturer’s instruction (line 167), which uses 30 minutes cutoff. Why 26 minutes was used here? Was the cuoff changed to decrease false-positives?

Line 295 and 327. Which positive control (gamma-irradiated or heat-inactivated) was used?

Line 340 and 346. How were these samples obtained?

Line 352. 5 copies per reaction? How was the copy number determined? RT-qPCR usually cannot quantify such a low copy number sample as it is out of range of the standard curve.

Line 501. The unit of viral loads should be mL not ul.

**********

6. PLOS authors have the option to publish the peer review history of their article (what does this mean?). If published, this will include your full peer review and any attached files.

If you choose “no”, your identity will remain anonymous but your review may still be made public.

**Do you want your identity to be public for this peer review?** For information about this choice, including consent withdrawal, please see our Privacy Policy.

Reviewer #1: **Yes: **Tim J. Dumonceaux

Reviewer #2: **Yes: **Yasufumi Matsumura

[NOTE: If reviewer comments were submitted as an attachment file, they will be attached to this email and accessible via the submission site. Please log into your account, locate the manuscript record, and check for the action link "View Attachments". If this link does not appear, there are no attachment files.]

While revising your submission, please upload your figure files to the Preflight Analysis and Conversion Engine (PACE) digital diagnostic tool, https://pacev2.apexcovantage.com/. PACE helps ensure that figures meet PLOS requirements. To use PACE, you must first register as a user. Registration is free. Then, login and navigate to the UPLOAD tab, where you will find detailed instructions on how to use the tool. If you encounter any issues or have any questions when using PACE, please email PLOS at figures@plos.org. Please note that Supporting Information files do not need this step.

# Author comment: Author response to Decision Letter 0

**DOI:** 10.1371/journal.pone.0268692.r002

4 May 2022

May 4, 2022

Dr. Kalendar

Academic Editor PLOS ONE

Manuscript: PONE-D-22-07620

Dear Dr. Kalendar,

 Thank you and the two reviewers for your thorough evaluation of our manuscript entitled “Development and Implementation of a Simple and Rapid Extraction-Free Saliva SARS-CoV-2 RT-LAMP Workflow for Workplace Surveillance”. We found the comments and suggestions by the reviewers most helpful and valuable for us to improve the manuscript, and we have done so accordingly. The following is a detailed list of our responses (in blue) to the comments raised and the changes we have implemented in the manuscript.

Journal Requirements:

When submitting your revision, we need you to address these additional requirements.

1. Please ensure that your manuscript meets PLOS ONE's style requirements, including those for file naming. The PLOS ONE style templates can be found at 

https://journals.plos.org/plosone/s/file?id=wjVg/PLOSOne_formatting_sample_main_body.pdf and https://journals.plos.org/plosone/s/file?id=ba62/PLOSOne_formatting_sample_title_authors_affiliations.pdf

We have revised the style requirements in the manuscript accordingly.

2. Thank you for stating the following in the Competing Interests section: 

"New England Biolabs (www.neb.com) has funded this study. All authors (ZL,JLB, BC, CVC, WEJ, KK, BWL, JB, RAM, CBP, GR, RJR, NAT, YZ and CKSC) are employees of New England Biolabs, manufacturer of LAMP and PCR reagents described in the manuscript." 

We note that you received funding from a commercial source: New England Biolabs

Please provide an amended Competing Interests Statement that explicitly states this commercial funder, along with any other relevant declarations relating to employment, consultancy, patents, products in development, marketed products, etc. 

Within this Competing Interests Statement, please confirm that this does not alter your adherence to all PLOS ONE policies on sharing data and materials by including the following statement: "This does not alter our adherence to PLOS ONE policies on sharing data and materials.” (as detailed online in our guide for authors http://journals.plos.org/plosone/s/competing-interests). If there are restrictions on sharing of data and/or materials, please state these. Please note that we cannot proceed with consideration of your article until this information has been declared. 

Please include your amended Competing Interests Statement within your cover letter. We will change the online submission form on your behalf.

The amended statement to include is the following: New England Biolabs (www.neb.com) has funded this study. ZL, JLB, BC, CVC, WEJ, KK, BWL, JB, RAM, CBP, GR, RJR, NAT, YZ and CKSC are employees and shareholders of New England Biolabs, manufacturer of LAMP reagents described in the manuscript. This does not alter our adherence to PLOS ONE policies on sharing data and/or materials.

3.Thank you for stating the following in the Acknowledgments Section of your manuscript: 

"This work was supported by New England Biolabs."

We note that you have provided funding information that is not currently declared in your Funding Statement. However, funding information should not appear in the Acknowledgments section or other areas of your manuscript. We will only publish funding information present in the Funding Statement section of the online submission form. 

Please remove any funding-related text from the manuscript and let us know how you would like to update your Funding Statement. Currently, your Funding Statement reads as follows: 

"New England Biolabs (www.neb.com) has funded this study. ZL, JLB, BC, CVC, WEJ, KK, BWL, JM, RAM, CBP, GR, RJR, NAT, YZ and CKSC are employees of New England Biolabs. The funders had no role in study design, data collection and analysis, decision to publish, or preparation of the manuscript."

Please include your amended statements within your cover letter; we will change the online submission form on your behalf.

We have removed the funding-related text from the acknowledgement section of the manuscript. The amended Funding Statement to include is the following: New England Biolabs (www.neb.com) has funded this study. 

4. We note that you have included the phrase “data not shown” in your manuscript. Unfortunately, this does not meet our data sharing requirements. PLOS does not permit references to inaccessible data. We require that authors provide all relevant data within the paper, Supporting Information files, or in an acceptable, public repository. Please add a citation to support this phrase or upload the data that corresponds with these findings to a stable repository (such as Figshare or Dryad) and provide and URLs, DOIs, or accession numbers that may be used to access these data. Or, if the data are not a core part of the research being presented in your study, we ask that you remove the phrase that refers to these data.

We have removed the phrase “data not shown” and either added new data (3 additional supplementary figures, S7 Fig, S8 Fig, and S9 Fig) to the manuscript to support the statement or removed the statement that refers to the data. 

5. Please review your reference list to ensure that it is complete and correct. If you have cited papers that have been retracted, please include the rationale for doing so in the manuscript text, or remove these references and replace them with relevant current references. Any changes to the reference list should be mentioned in the rebuttal letter that accompanies your revised manuscript. If you need to cite a retracted article, indicate the article’s retracted status in the References list and also include a citation and full reference for the retraction notice.

We have added the 3 references suggested by the reviewers, namely references #37, 38 and 61. We have also added 2 references that relate to the use of the kappa analyses, #39 and #40. The reference list has been updated accordingly. 

Reviewer:1

In this manuscript, the authors have developed and validated a saliva-based nucleic acid amplification test (RT-LAMP) that offers speed and convenience compared to the typical NAAT that is employed for SARS-CoV2 detection, RT-qPCR. The authors optimized a buffer for virus inactivation and RNA stabilization from saliva, and implemented fluorescence-based and colorimetric detection systems for determining the results of the reaction. Authors determined the major validation parameters for a diagnostic test, including analytical sensitivity (not specificity), and diagnostic sensitivity and specificity compared to the RT-qPCR test. The authors employed the test for the purposes of surveillance at a workplace setting, and screened a large number of samples (>30k) over the course of nearly a year. Authors conclude that the RT-LAMP can offer a rapid, convenient, and relatively inexpensive means to monitor staff for infection in order to mitigate workplace spread of covid. Overall, this is a comprehensive and well-written description of a fast and convenient means to detect SARS-CoV2 in clinical samples that can be challenging (saliva), but offer many advantages compared to deep nasal swabs. There is really not much to criticize in this work. I offer below some minor comments and suggestions for improvement.

Materials and Methods

1. Line 187: The authors should mention here that orange-colored reactions (which in my experience tend to occur when target NA levels are low, and the reaction is just beginning to turn positive when the assay is stopped) were considered negative. This is mentioned much later on in the Results section (lines 402, 404, and Table 2), but should be mentioned here for clarity as well.

As suggested, we have revised the sentence to include “orange”: “whereas a Tt > 26 minutes or N/A, and a post reaction color of pink or sometimes orange, indicated no detection”.

Results

2. Line 261 and 278 (Figure 2 legend): I suggest that the authors change the title of this section – “diagnostic sensitivity” typically refers to the performance of a newly developed assay compared to a previously established “gold standard” assay, so this is what I was expecting this section to describe. Instead, that comes later. Perhaps something like, “Impact of saliva input on RT-LAMP performance” or something similar might be suitable.

As suggested, we have revised the figure and section titles to : Impact of saliva input on RT-LAMP performance.

3. Lines 322-323: This section describes the determination of the analytical sensitivity (LOD) of the RT-LAMP reaction. The authors should specify how the LOD was defined – this is typically specified as the C95, or the concentration of analyte that results in a positive reaction in 95% of assays (see Burd EM Clin. Microbiol. Rev. 23, 550-576, doi:10.1128/cmr.00074-09 (2010)). Moreover, the authors seem to have determined LOD empirically, but there are more accurate means of calculating the C95 for which the authors already have the data – eg see Klymus, K. E. et al. Environ. DNA 2, 271-282, doi:https://doi.org/10.1002/edn3.29 (2020). Please consider expressing the analytical sensitivity data using one of the mathematical models described therein, or alternatively using probit analysis to provide a more precise estimate of the LOD of the assay.

As suggested, we used the method described by Klymus et al. to calculate the C95 of the LOD and included the data in the results. Accordingly, we have added 2 new references to the manuscript.

4. Lines 339-345: It was nice to see the Dsp and Dsn of the RT-LAMP assay compared to the RT-qPCR assay, although the number of samples analyzes was rather small. The results are overall rather similar to the values obtained using LAMPore on much larger sample sizes (Ptasinska, A. et al. Clinical microbiology and infection 27, 1348.e1341-1348.e1347, doi:10.1016/j.cmi.2021.04.008 (2021). This reference should be cited in the Discussion. The results obtained here are comparable and vastly less expensive compared to LAMPore, which is only reasonable to perform on large numbers of samples and is therefore not suitable for point-of-care applications.

We have incorporated the comments into the discussion and added the suggested reference to the manuscript.

5. Lines 396-406 and Table 2: These results could be analyzed using the kappa statistic, which is ideally suited to assessing assay performance with a subjective aspect like color development. Please consider adding a kappa calculation to demonstrate the operator agreement.

As suggested, we have analyzed the data obtained from the color and fluorescence output across all operators to demonstrate the high concordance as this was the focus of the study. We used the kappa statistic to demonstrate the high concordance (kappa value =0.999, 95% CI 0.966-0.999) between the 2 readouts. We have incorporated the results in the main text and added a new supplementary figure S6 Fig. Two references describing the statistical methods have also been added to the reference section. 

Reviewer 2:

Li et al. developed an extraction-free, direct RT-LAMP protocol and validated for screening of employees. The purpose and flow of the study is well defined and methods were around technical standard. Modifications that clarify some technical details/objective assessments that are important as a scientific paper are needed before consideration for publication.

The study was funded by a company and all authors belonged to that company. The study focused on the company’s product. In this situation, is it good for the authors to simply declare that the funder had no role in conducting the study?

We have addressed this point and modified the competing interest statement as per journal policies. See above. 

Line 52. What was the limit of detection of the developed workflow? 50 copies/uL of samples is far less sensitive than the commercially available RT-qPCR-based assays (usually they show LODs around 1 copies/ul).

As per the recommendation of reviewer #1, we have used a C95 LOD calculation to determine a value of 39 copies/�L of biofluid (saliva). We acknowledge that this value is lower that some results reported using purified RNA from saliva/nasal swabs and RT-qPCR. However, our comparison demonstrates we can detect all positive saliva samples with a RT-qPCR Cq value less than 34, which has been used as a cut-off value by some commercial tests. 

Line 56-58. This conclusive statement is not based on the presented results. How rapidly were these infected cases diagnosed and isolated? At least describe turn-around time and time to isolation. How was the viral loads of these cases and how many cases were asymptomatic? I recommend the authors to make a conclusive statement for the developed assay, not what is speculated and is limited to workplace surveillance.

The turnaround time was ~2 hours and employees were isolated immediately if a positive test result was obtained. We believe this practice reduced the probability of viral spread in the workplace. Recording viral loads and onset of symptoms for each employee was beyond the scope of this study. Therefore, we have removed the statement from the abstract.

Line 119. Please elaborate RNA extraction and RT-qPCR. How many samples were provided?

For RNA extraction, the ThermoFisher King Fisher Flex automated RNA extraction system with the MagMax viral pathogen kit, was used according to protocol. This has been incorporated into the manuscript.

For RT-qPCR, the ThermoFisher TaqPath COVID-19 Combo Kit for N, Orf1ab, S genes (A47814), a kit authorized under EUA for SARS-CoV-2 detection, was used. This has been incorporated into the manuscript.

30 positive and 30 negative samples were randomly selected from a total of 90 confirmed (either negative or positive) samples, based on volume available to allow for repeat testing. 

Line 139. What was the volume of sample used for RNA purification?

For comparison studies, RNA was purified from 200uL of saliva using the Monarch® Total RNA Miniprep Kit (NEB, Cat# T2010. This information has been incorporated into the manuscript.

Line 163. Which realtime PCR system was used for fluorescence detection?

A Bio-rad CFX96 instrument was used for RT-qPCR fluorescence detection. This information has been incorporated into the manuscript.

Line 197-203 is duplicate of line 130-142.

We have deleted the duplicated text at second mention.

Line 279. Which centrifugation conditions were used?

No centrifugation was involved. Saliva naturally settles into supernatant and sediment. These fractions were used as described. 

Line 295. Is 50 copies/ul concentration used for reaction=100 copies/reaction? Please clarify for understanding. If so, this is equevalent to 25 copies/ul=25000 copies/mL of inactivated saliva. Even with this high concentration, 1.4% of samples were tested negative, indicating low sensitivity of the assay and this is supported by LOD analysis.

Saliva samples were spiked with 50 copies/µL of SARS-CoV-2 virus. Each spiked saliva sample was then mixed with an equal volume of 2X lysis buffer (therefore equivalent to 25 copies/µL) to generate a saliva lysate. 2 µL (50 copies) of saliva lysate was added into the LAMP reaction. Accordingly, 98.6% of samples tested positive at 50 copies/µL of original saliva. 

We have amended the text in the Materials and Methods to clarify this point: “For each RT-LAMP reaction, 2 µL of treated saliva sample, corresponding to 1 µL of neat saliva, was used in a 20 µL reaction”. 

Line 297. The RT-LAMP reaction was performed according to manufacturer’s instruction (line 167), which uses 30 minutes cutoff. Why 26 minutes was used here? Was the cuoff changed to decrease false-positives?

The text has been modified to reflect that a slightly modified protocol was used. This includes using a 20 µL (not 25 µL) reaction volume and total reaction time of 35min (not 30min). When fluorescence is used as a readout, a cut-off Tt value of 26 minutes was used to differentiate positive/negative reactions and eliminate background signals observed in very few saliva samples. 

Line 295 and 327. Which positive control (gamma-irradiated or heat-inactivated) was used?

Heat-inactivated virus was used. This information has been included in the text.

Line 340 and 346. How were these samples obtained?

Samples were obtained from consented individuals using the Mirimus SalivaClear Collection Kit (cat# 800100) according to the SalivaDirect FDA EUA ( EUA202097). Samples were de-identified and provided to NEB, under the SUNY Downstate IRB protocol #1603504.

Line 352. 5 copies per reaction? How was the copy number determined? RT-qPCR usually cannot quantify such a low copy number sample as it is out of range of the standard curve.

Twist RNA with copy number provided by the manufacturer was used to generate a standard curve. In RT-qPCR reactions (performed in triplicate) using 5 copies of twist RNA as input, Cq values of 34.6, 34.5, and 34.5 were obtained for the SARS-CoV-2 N1 gene. Therefore, we estimated the Cq value of 35 corresponds to ~5 copies of virus RNA.

Line 501. The unit of viral loads should be mL not ul.

The value is cited in an included reference (Huang et al). The paper cited the viral copy number in µL rather than mL as the amount in biofluid. We added ‘biofluid’ to the text to accurately cite the reference and clarify the context.

 We believe these revisions have improved the manuscript immensely and hope that you and the reviewers will consider it suitable for publication of PLOS ONE. 

With my best regards, 

Clotilde Carlow, Ph.D

Scientific Director 

Division of Genome Biology

Attachment Submitted filename: Response to Reviewers 05_04_22.docx Click here for additional data file.

# Editor report: Decision Letter 1

**DOI:** 10.1371/journal.pone.0268692.r003

**Authors:** Ruslan Kalendar

6 May 2022

Development and implementation of a simple and rapid extraction-free saliva SARS-CoV-2 RT-LAMP workflow for workplace surveillance

PONE-D-22-07620R1

Dear Dr. Carlow,

We’re pleased to inform you that your manuscript has been judged scientifically suitable for publication and will be formally accepted for publication once it meets all outstanding technical requirements.

Within one week, you’ll receive an e-mail detailing the required amendments. When these have been addressed, you’ll receive a formal acceptance letter and your manuscript will be scheduled for publication.

An invoice for payment will follow shortly after the formal acceptance. To ensure an efficient process, please log into Editorial Manager at http://www.editorialmanager.com/pone/, click the 'Update My Information' link at the top of the page, and double check that your user information is up-to-date. If you have any billing related questions, please contact our Author Billing department directly at authorbilling@plos.org.

If your institution or institutions have a press office, please notify them about your upcoming paper to help maximize its impact. If they’ll be preparing press materials, please inform our press team as soon as possible -- no later than 48 hours after receiving the formal acceptance. Your manuscript will remain under strict press embargo until 2 pm Eastern Time on the date of publication. For more information, please contact onepress@plos.org.

Kind regards,

Ruslan Kalendar

Academic Editor

PLOS ONE

# Editor report: Acceptance letter

**DOI:** 10.1371/journal.pone.0268692.r004

**Authors:** Ruslan Kalendar

17 May 2022

PONE-D-22-07620R1 

Development and implementation of a simple and rapid extraction-free saliva SARS-CoV-2 RT-LAMP workflow for workplace surveillance 

Dear Dr. Carlow:

I'm pleased to inform you that your manuscript has been deemed suitable for publication in PLOS ONE. Congratulations! Your manuscript is now with our production department. 

If your institution or institutions have a press office, please let them know about your upcoming paper now to help maximize its impact. If they'll be preparing press materials, please inform our press team within the next 48 hours. Your manuscript will remain under strict press embargo until 2 pm Eastern Time on the date of publication. For more information please contact onepress@plos.org.

If we can help with anything else, please email us at plosone@plos.org. 

Thank you for submitting your work to PLOS ONE and supporting open access. 

Kind regards, 

PLOS ONE Editorial Office Staff

on behalf of

Professor Ruslan Kalendar 

Academic Editor

PLOS ONE
